# Differences in family caregiver experiences and expectations of end-of-life heart failure care across providers and settings: a systematic literature review

**DOI:** 10.1186/s12913-023-09241-w

**Published:** 2023-05-03

**Authors:** Alessandro Valleggi, Claudio Passino, Michele Emdin, Anna Maria Murante

**Affiliations:** 1 Fondazione Gabriele Monasterio, Pisa, Italy; 2 Interdisciplinary Research Center Health Science, Scuola Superiore Sant’Anna, Pisa, Italy; 3 Management and Health Lab - Institute of Management, Scuola Superiore San’Anna, Pisa, Italy

**Keywords:** Caregivers, Service experience, Care setting, Care provider, Terminal care, Heart failure

## Abstract

Heart failure impacts patients’ quality of life and life expectancy and significantly affects the daily behaviours and feelings of family caregivers. At the end-of-life, the burden for family caregivers depends on their emotional and sentimental involvement, as well as social costs.

**Objectives:** The aim of this work is to determine whether and how family caregivers’ experiences and expectations vary in relation to the places of care and teams involved in heart failure management.

**Methods:** A systematic literature review was conducted, by screening manuscripts dealing with the experience of Family Care Givers’ (FCGs) of patients with Advanced Heart failure. Methods and results were reported following the PRISMA rules. Papers were searched through three databases (PubMed, Scopus and Web of Science). Seven topics were used to synthetize results by reporting qualitative information and quantitative evidence about the experience of FCGs in places of care and with care teams.

**Results:** Thirty-one papers, dealing with the experience of 814 FCGs, were selected for this systematic review. Most manuscripts came from the USA (*N* = 14) and European countries (*N* = 13) and were based on qualitative methods. The most common care setting and provider profile combination at the end of life was home care (*N* = 22) and multiprofessional teams (*N* = 27). Family caregivers experienced “psychological issues” (48.4%), impact of patients’ condition on their life (38,7%) and “worries for the future” (22.6%). Usually, when family caregivers were unprepared for the future, the care setting was the home, and there was a lack of palliative physicians on the team.

**Discussion:** At the end-of-life, the major needs of chronic patients and their relatives are not health related. And, as we observed, non-health needs can be satisfied by improving some key components of the care management process that could be related to care team and setting of care. Our findings can support the design of new policies and strategies.

**Supplementary Information:**

The online version contains supplementary material available at 10.1186/s12913-023-09241-w.

## Introduction

This review aims to explore the experiences and expectations of family caregiving (FCGs) of heart failure (HF) patients at the end of life and explores whether they vary in relation to the places of care (e.g., hospital, home care, hospice) and the profile of professionals involved in the care (e.g., palliative care (PC) providers, cardiologists, family doctors, nurses).

HF is a chronic, progressive condition and is the final stage of all cardiovascular diseases [1]. Worldwide, nearly 26 million people are affected by HF, generating a considerable global economic burden for healthcare systems of approximately $31 billion per year [2]. Together with cancer, HF is one of the most challenging conditions to manage for healthcare providers due to the ageing population and the complexity of various associated comorbidities. Indeed, with advancements in treatments and strategies of care, people live longer with progressive worsening of general conditions and related symptoms, leading patients to live with the disease for up to several years. This is related to several medical, psychosocial and economic issues, from the very first stages of disease to the more advanced conditions until the last phase termed “end of life”. Long-term prognosis remains poor, with 50% of patients dying within five years of diagnosis [3] and with many patients experiencing progressive functional and physical decline and consequent multiple hospitalizations. Although care management for chronic HF is well defined and although there is substantial consensus within professional communities on its effectiveness, uncertainty about care prevails for end-stage HF and the end-of-life period [4] and for the provision of PC. Currently, PC is recommended for managing HF care by the most important cardiology associations [5–7]. A position statement of the European Society of Cardiology Heart Failure Association [8] affirmed that “successful PC must involve shared care through a multidisciplinary approach. Patients and their caregivers should be able to easily communicate with primary care, specialist PC services and the specialized advanced HF service, according to the resources of each centre. Aging, co-morbid conditions, end-organ damage, cognitive impairment, frailty and limited social support complicate HF management, and PC should address each of these components”.

This complex assistance approach often requires the regular participation of unpaid FCGs. Until the early 2000s, scholars minimally focused their research on FCGs’ role, needs and experience. Only in the last decade has the role of FCGs in HF management been progressively recognized as relevant by both scholars [9] and medical associations [10]. In addition to the social and economic costs generally shouldered by FCGs of patients at the end-of-life stage [11], significant stress affects FCGs’ lives due to the daily activities required of them to make healthcare providers’ care management effective (i.e., managing medications, helping communicate with healthcare providers, coping with symptoms management) [12, 13]. Hence, at the end of life, care management should also address all the physical, psychological, social and spiritual needs of both patients and their families [14].

This holistic approach in taking care of patients’ and families’ needs is typical of PC and allows us to achieve goals such as improving quality of life, symptoms and patient-clinician communication [15–17] for HF patients and their families [18–20]. Despite the value derived from adopting this approach, PC services are still rarely used to fulfil the needs of HF patients and their relatives at the end-of-life stage [21–24], and when patients are referred to PC, late activation of PC is frequently observed, with decreasing positive benefits for patients and FCGs [25].

Due to the relevant role played by informal FCGs, the burden of disease management they experience and the limited access of HF patients to PC, this manuscript aims to systematize the existing literature on the experiences of FCGs of HF patients to increase policymakers’ and practitioners’ awareness of the FCG experience, expectations and needs. Specifically, this systematic literature review aims to answer the following research question: *“Do FCGs’ experiences and expectations vary in relation to the setting of care and the care provider(s)?”.*

### Setting of care, care providers and family caregivers

The uncertainty of HF prognosis and progressions leads to different points of view regarding the adaptation of the therapies provided as well as the identification of the appropriate setting of care and care provider. While in the last decade, researchers have worked to analyse the world of FCGs in advanced HF, no attention has been given to how the combination of the care setting and care provider profile could influence FCGs’ experiences. No literature focusing on this specific theme exists and we believe that further knowledge could support health professionals and health systems to optimize HF management, define health care policies and consequently allocate adequate resources to support FCGs while optimising HF care.

#### Combination of the care setting and provider profile

Defining the appropriate setting of care in advanced HF management is relevant: patients usually live in their homes, with hospitalizations during decompensation, and only a minority of patients may be assisted in long-term care (LTC) facilities, nursing homes or hospices. Historically, hospices have been underutilized for HF patients. Even though in 2012 a study showed that the admission rate for HF increased from 19 to 40% [26], the utilization of hospice services was still < 10% [27]. This low utilization rate is due to the uncertain trajectory of disease and cultural barriers that make it difficult to plan care [4].

The choice of the place of care may be influenced by patient- and FCG-specific needs. Preferences may change over the evolution of HF and differ between patients and their FCGs, resulting in incongruences and conflicts [28]. In a randomized control trial, Brännström and colleagues [29]demonstrated that “person-centred care combined with active heart failure and PC at home has the potential to improve quality of life and morbidity in patients with severe chronic heart failure”. That intervention was provided in home care units by a multidisciplinary team composed of specialized nurses (in HF and PC), cardiologists and PC specialists.

Focusing on those who should be in charge of end-of-life care, Rogers and colleagues [30] showed that PC intervention can produce significant benefits for quality of life when care is provided by HF nurses and when PC specialists and HF-specialized cardiologists work together. Additionally, Daley and colleagues [31] evaluated cost-effective and sustainable collaboration between community-based HF nurse specialists and specialist PC services.

A multidisciplinary approach is recommended even though large variability exists in HF management programs across Europe [5]. The majority of existing programs have HF nurses and physicians (cardiologists and family doctors) involved on their teams [32]. The dimension of involvement of PC specialists may be underestimated: data from an American survey in 2016 [33] stated that there was a PC specialist for every 1200 persons living with HF. After nurses and physicians, physiotherapists (33%), social workers (23%) and pharmacists (19%) are most commonly involved on HF teams. This type of multiprofessional team has become the most diffused model in Europe, particularly in the UK [5].

#### Family caregivers’ experiences and expectations

As the disease advances, patient management becomes more complex, and the role of FCGs becomes increasingly crucial and increasingly stressful, resulting in anxiety, depression and social isolation for FCGs [34–36]. Generally, the FCG role is assessed by the use of indicators of FCG wellbeing as well as measures of end-of-life care, among others [37]. In recent years, researchers have shifted the focus of their insights to existing models of support for FCGs, with heterogeneous results in terms of positive effects on care and the improvement of outcomes [38, 39]. Mcllfatrick and colleagues also showed that FCGs have unmet needs and feel unprepared for the future and that they lack emotional support and advanced care planning with professionals [40]. In 2017, one of the first literature reviews [41] on FCG needs revealed that inadequate communication with healthcare providers is one of the most important concerns for FCGs. The authors also examined FCGs’ psychosocial needs in terms of care burden and emotional distress with the results being similar for other diseases. The above evidence demonstrates an increasing need to educate FCGs on coping strategies to reduce FCG burden and to increase cooperation with professionals. These results allow us to perceive what it may mean for FCGs to live with HF patients at the end of life, but this evidence still falls short in providing direction on the care setting and care provider combination that best fulfils FCG needs.

## Methods

In accordance with the PRISMA 2020 item checklist [42], here we report the methods used for our literature review through the following steps. *Eligibility criteria:* Our review work focused on studies: 1) dealing with the experience of FCGs of adult individuals with advanced HF; 2) published up to December 31, 2021; 3) written in English; and 4) peer-reviewed (selection criteria). Studies involving FCGs of HF patients with additional disease were included, while studies including patients with different diseases, even if some HF patients were present, were excluded. Previous literature review papers were also excluded. Studies have been grouped by setting of care (Home care, Hospice, Hospital, Long Term Care) and composition of the care team (Mono-professional, Multi-professional and Multi-professional with PC specialists).

### Information sources and search strategies

Papers were extracted from three different electronic databases (PubMed, Scopus and Web of Science), by applying an algorithm including terms dealing with Heart Failure, end of life and palliative care, family caregiving. Supplemental 1 reports the list of keywords include in the search algorithms used for each database. The keywords were searched in the abstracts, titles, and keywords.

### Selection process

A four-member research team took part in the literature review processes, from the study design, the definition of search and analysis strategies, to the reading of the abstracts and full articles. Any doubts they met during the screening work were discussed together. The four research members proceeded with the abstract reading to confirm that a part or all the abstracts fulfilled the inclusion criteria and dealt with *FCGs’ experience and expectations* of *adult HF patients*, at the *end of life.*

**Fig. 1:**
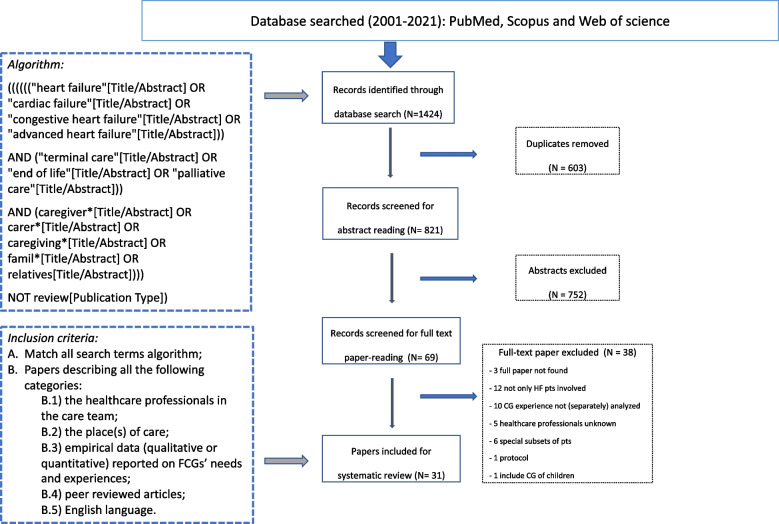
PRISMA diagram

For papers approved in this first screening stage, the corresponding full texts were then read to state the factual presence of content dealing with: FCG experiences and expectations, setting of care, and professional profile of the team members who were responsible for the patients’ care. Literature reviews, case reports and articles that analyzed only professional carers or patients without reporting on FCG experiences were excluded. Furthermore, studies involving patients with other conditions (i.e., COPD, cancer, dementia) or special groups of patients (i.e., patients with left ventricular assist devices and transplanted patients) were excluded from the analysis to avoid possible confounding results (Fig. 1).

### Data collection process

For eligible papers, data on country, study design and method(s), sample and variable/measure(s) considered were collected to report the studies’ characteristics. For each paper, information on the following elements were collected: setting of care, team composition and FCGs’ experiences and expectations. These elements were analyzed and later grouped by homogeneous topics identified through a quantitative content analysis and ordered for the setting of care and the composition of the care team.

### Synthesis methods

A table was created to capture information on the characteristics of studies, frequencies of combination of settings of care and team compositions and quality of FCG experiences. Graphical tools were used to synthetize and map the recurrence of topics across the setting of care and the type of teams involved in care provision.

## Results

The search for papers was conducted in three databases, resulting in a total of 1424 papers. After removal of 603 duplicates, 821 abstracts were screened, and 69 articles were determined to be suitable for full reading. At the end of the screening process, a total of 31 articles passed the critical quality appraisal according to the CASP checklist [43], were determined to be suitable for full analysis and were included in the review (Fig. 1).

### Study characteristics and variables measured

Table 1 reports the key details of the papers, including the study design, method, country, sample, and variables/measures. The studies were mainly conducted in the USA [44–57], UK [58–67] and Canada [68–70]. Ireland and Sweden were the only two European countries with published papers, showing that this topic is still rarely addressed by researchers in Europe.

**Table 1:** Characteristics of studies and FCG measures

Study	Design	Method	Country	FCG’s Sample	Variable/measures
Hupcey et al. 2010 [44]	Instrumental case studies. Narrative interview	Qualitative	USA	5	Uncertain trajectory of HF; living with a slow decline in HF; experience of a hospice care
Alonso et al. 2017 [45]	Descriptive interview	Qualitative and quantitative	USA	80	Disease severity; disease terminality
Imes et al. 2011 [46]	Semistructured interview	Qualitative	USA	14	Experience of FCG with HF; experience of FCG with providers; patient's experience as perceived by partner
Bakitas et al. 2017 [47]	Feasibility study	Quantitative	USA	48	Comparison between ENABLE-CHF-PC intervention in two sites for patient outcomes and FCG outcomes (BCOS, HADS anxiety and depression, physical and mental health, MBCB, stress burden and mental burden, PAC self affirmation, outlook on life)
Buck et al. 2013 [48]	Semistructured interview	Qualitative	USA	7	Hospice experience and utility of the book
Neuwirth et al. 2012 [49]	Videoethnography	Qualitative	USA	3	Qualitative description of utility of video in managing pts an CG (very few about FCG)
Hupcey et al. 2011 [50]	Semistructured interview (grounded theory design)	Qualitative	USA	45	Financial, psychosocial, physical issues
Retrum et al. 2013 [51]	Semistructured interview	Qualitative	USA	17	congruence and incongruence between dyads members
Metzger et al. 2013 [52]	Semistructured interview	Qualitative	USA	16	knowledge of PC; role of PC; PC and hospice
Schwarz et al. 2012 [53]	Retrospective collection of interviews	Qualitative	USA	20	Role of PC consultation
Metzger et al. 2013 [54]	Semistructured interview	Qualitative	USA	16	Knowledge of PC; role of PC; conflation between PC and hospice
Dionne-Odom et al. 2014 [55]	Two-phase formative evaluation study	Quantitative and qualitative	USA	11	FCG burden; QoL of FCG. Measures with scales
Mcmillan et al. 2007 [56]	Retrospective chart review	quantitative	USA	37	In hospice psychosocial issues measured with CES-D
Alonso et al. 2018 [57]	Descriptive interview	Qualitative	USA	23	caregiver resources, role management, caregiver-parent relationships, filial responsibility, and personal benefits and challenges
Aldred et al. 2005 [58]	Narrative interview	Qualitative	UK	10	Impact on everyday life; impact on relationships patient-FCG); professional support; concern about the future; lack of time from doctors (FD and cardiologist)
Stocker et al. 2017 [59]	Semistructured interview (grounded theory design)	Qualitative	UK	3	Prognosis/diagnosis; future
Boyd et al. 2004 [60]	Semistructured interview	Qualitative	UK	20	Physical problems; psychosocial issues; organization of care; end-of-life
Harding et al. 2008 [61]	Semistructured interview	Qualitative	UK	11	Symptoms management; future care; living without infos; barrier to communication
Browne et al. 2014 [62]	Semistructured interview	Qualitative	UK	20	knowledge deficit, difficulty in accessing health and social care support, barriers to optimal care
Ross et al. 2015 [63]	Semistructured interview	Qualitative	UK	8	Spiritual needs
Leeming et al. 2014 [64]	Semistructured interview	Qualitative	UK	12	Social isolation; coping strategies; End-of-life worries; family roles
Simmonds et al. 2015 [65]	Longitudinal, patient-led ethnography	Qualitative	UK	9	How FCG perceive living with HF and key events in illness
Small et al. 2009 [66]	Semistructured interview	Qualitative	UK	20	Analysis of period prior to death; hospital staying; the bereavement
Chester et al. 2021 [67]	Focus interview	Qualitative	UK	4	perceptions of referral to palliative care, key components of the new service that were deemed helpful, and unhelpful in terms of care
Alvariza et al. 2017 [71]	Semistructured interview	Qualitative	Sweden	14	Impact on FCG of pts conditions; FCG state of mind and condition
Brannstrom et al. 2007 [72]	Narrative interview	Phenomenological-hermeneutic method. Qualitative	Sweden	4	Responsibility of care (physical, emotional)
Ng et al. 2017 [73]	Randomized controlled trial	quantitative	Hong-Kong	84	FCG burden with ZBI scale For PT QoL; symptom burden
Kaasalainen et al. 2013 [68]	Descriptive interview	Qualitative	Canada	7	Living with restrictions of HF and comorbidities; decision-making about end-of-life; communication
Schulz et al. 2017 [69]	Semi-structured interviews	Qualitative	Canada	209	Psychosocial aspects of death and dying
Im et al. 2019 [70]	Descriptive interview	Qualitative	Canada	19	understanding of illness, uncertainty and end‐of‐life communication
Fitzsimons et al. 2019 [74]	Descriptive interview	Qualitative	Ireland	30	communication with professionals, knowledge about future

Only five papers used a quantitative approach [45, 47, 55, 56, 73], while the majority used a qualitative approach by mainly using in-depth semi-structured interviews and narrative interviews. Out of the 31 papers, the majority had small samples, with only five papers including > 40 participants [45, 47, 50, 69, 73].

Most studies involved FCGs of patients with a diagnosis of NYHA Class III/IV or AHA Stage C/D HF, with some exceptions [44, 47–50, 52, 56, 59, 65, 68], while predicted survival (when reported) varied across studies [45–48]. Generally, the studies directly or indirectly considered the role of PC in advanced HF care, and seven studies only referred to end of life without a specific reference to PC [47, 50, 52, 66, 69, 70, 73]. The definition of PC was homogeneous across the studies when it was reported [50, 52, 54, 58, 60, 61, 64, 67, 71, 73] and was based on the WHO definition [14].

The FCG experiences and expectations can be tracked back to seven topicss, dealing with: “impact of the patient condition on FCGs” [45, 46, 49, 55–58, 60, 64, 68, 70, 71, 74], “psychological issues of FCGs” [47, 50, 53, 55–57, 60, 63, 64, 66, 69, 71–74], “relationship with patients” [51, 57, 58, 61, 70, 73, 74], “relationship with professionals” [46, 48, 52, 57, 58, 61, 62, 65–68, 70, 74], “worries and plans for the future” [44, 58, 59, 61, 67, 68, 70, 74], “role of PC” [52, 54, 67, 74] and “financial aspects” [50, 58, 72, 73].

### Care setting and team members

As Tables 2, 3 and 4 show, compared to other setting-based studies that were mostly published after the 2010s, FCG experience in the home care setting was continuously and fully investigated since 2004. Home care was the most common place of care in 21 of 31 papers [44, 46, 47, 49–51, 53, 55, 57–61, 63–65, 69, 70, 72–74], with multidisciplinary and multiprofessional teams involved, with some exceptions [44, 46, 50, 72, 74]. Depending on the study, teams were composed differently: cardiologists and nurses (57.1%) [47, 51, 53, 57–59, 61, 63, 65, 69, 70, 73] or palliative specialists (38.%) [53, 55, 60, 61, 63, 69, 70, 73].

**Table 2:** Distribution of professional profiles and places of care across the 31 reviewed papers

	HOME CARE	HOSPICE	HOSPITAL	HOSPITAL & HOSPICE	HOSPITAL & HOME CARE & NURSING HOME	LTC FACILITIES	HOSPITAL & HOSPICE & HOME	TOTAL
Palliative physician	25.81	6.45	9.68	3.23	0.00	0.00	0.00	45.16
Cardiologist	51.61	3.23	9.68	3.23	3.23	6.45	3.23	80.65
Family Doctor	22.58	0.00	3.23	0.00	0.00	0.00	0.00	25.81
Nurse	51.61	6.45	3.23	0.00	3.23	6.45	3.23	74.19
Social worker	12.90	9.68	0.00	0.00	0.00	3.23	0.00	19.35
Pharmacist	3.23	0.00	3.23	0.00	0.00	3.23	0.00	9.68
Others	6.45	0.00	3.23	0.00	0.00	0.00	0.00	9.68
Total	67.74	6.45	9.68	3.23	3.23	6.45	3.23

**Table 3:**
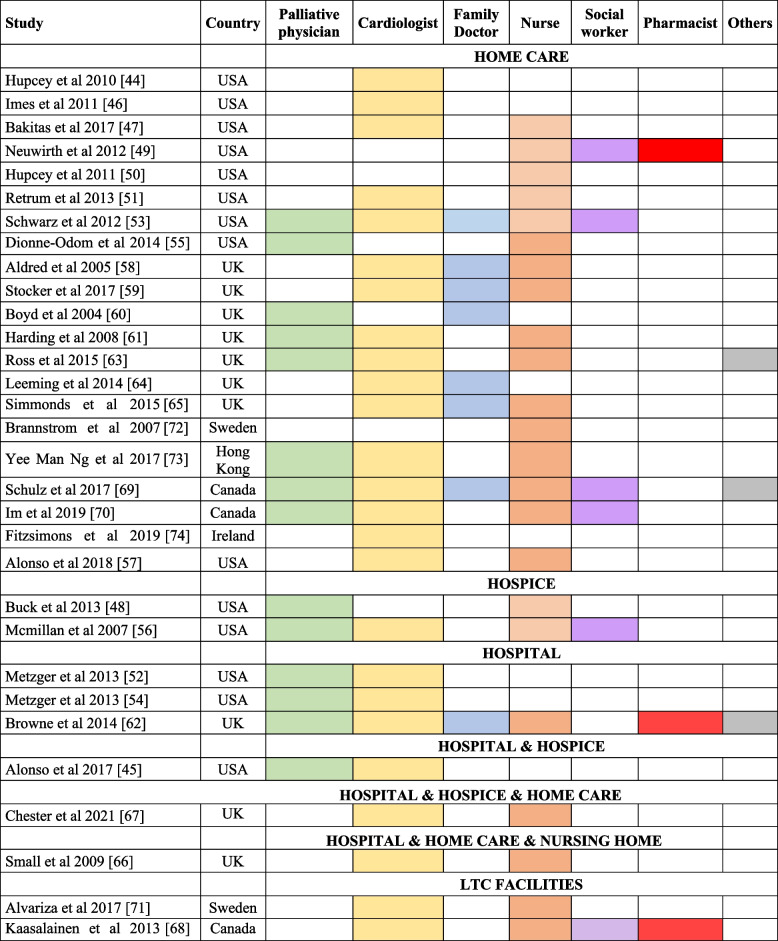
Care setting and provider profile’s combinations

Shaded cells report the provider’s presence

**Table 4:** Main findings on FCGs’ experience and needs by setting and provider of care

Study	Place of care	Team members	Main findings about FCGs experience	Main experience topics
Hupcey et al. 2010 [44]	Home care	Cardiologist	FCGs not prepared, did not understand the end was near; responsibility of care, financial and emotional strain of the experience; relief and sadness waiting for the death	Worries and plans for the future; Financial aspects; Psychological issues
Imes et al. 2011 [46]	Home care	Cardiologist	*Experience*: Negative impact on daily relationship with patients (not going out as usual, stress and challenge in everyday life, avoid discussion on future, sometimes more communication between partners), impact on FCGs (emotional impact, change in routine, FCGs helped by thinking of themselves); lack of information exchange between FCGs and providers (little information on prognosis and confusion); FCGs see patients negatively approaching the end-of-life (struggle with symptoms, living day-to-day with uncertainty, not talking about death, no advanced care planning, always hoping for the best, some not caring about the future) Expectations: have more info to prepare for the future, on disease, symptoms	Relationship with professionals or services; impact of patients’ condition on FCGs
Fitzsimons et al. [74]	Home care	Cardiologist	Experience: poor communication with professionals and little understanding of the future, living with uncertainty; lack of service provision and understanding of PC services. Negative feelings about living with their loved ones, fear, loneliness, sadness; their loved ones’ complaints about dying alone	Psychological issues; Relationship with professionals or services; Worries and plans for the future; Impact of patients’ condition on FCGs; Relationship with patients; Role of PC
Leeming et al. 2014 [64]	Home care	Cardiologist, Family doctor	*Experience*: isolation leads to psychosocial problems in FCGs; pivotal role of religious support; lack of knowledge about future; FCGs worry about health of patient and vice versa; lack of communication about fears or overprotection	Psychological issues; Impact of patients' condition on FCGs
Aldred et al. 2005 [58]	Home care	Cardiologist, Family doctor, nurse	*Experience*: Isolation in home, activities curtailed; FCGs key role in physical and emotional support, struggle and frustration with coping with patient needs, FCGs have their own physical problems; lack of time from doctors (Family Doctor and cardiologist); preoccupation and fear about the future *Expectations*: Need more time to talk with doctors, need to discuss disease, future and prognosis	Relationship with professionals or services; Impact of patients’ condition on FCGs;
Stocker et al. 2017 [59]	Home care	Cardiologist, Family doctor, nurse	*Experience*: unclear understanding of diagnosis, no willingness to know prognosis; talk about death in general terms; uncertainty of future but looking at improvements in future	Worries and plans for the future
Simmonds et al. 2015 [65]	Home care	Cardiologist, Family doctor, nurse	*Experience*: fragmented management, lack of continuity; lack of nurse services in the community; difficulty in contacting the hospital; lack of communication about prognosis and future	Relationship with professionals or services
Bakitas et al. 2017 [47]	Home care	Cardiologist, nurse	*Experience*: improvements in BCOS, HADS-depression, global mental health and MBCB score	Psychological issues
Retrum et al. 2013 [51]	Home care	Cardiologist, nurse	*Experience*: congruence in end-of-life issues, in planning for the future, managing illness for some dyads and incongruence for others. Incongruence in self-care. Absence of communication between patients and FCGs. Incongruence associated with distress and tension, congruence with solidarity. Dyads involving a spouse or partner had more emotional investment. Older couple means higher level of acceptance *Expectations*: need of a care team	Relationship with patients
Alonso et al. 2018 [57]	Home care	Cardiologist, nurse	*Experience:* feel alone, difficulties in caring for their loved ones and for themselves, they sacrifice their life for them, lack of time for their activities. Positive effect on FCGs’ style of life, healthy behavior, no smoking etc Expectancies: needs for every day support for life and spiritual needs	Psychological issues; Impact of patients’ condition on FCGs; Relationship with patients
Schulz et al. 2017 [69]	Home care	Cardiologist, palliative physician, Family doctor, nurse, other specialists, social worker	*Experience*: avoid talking about death, frustration and sadness, but at the same time FCGs understand and accept it	Psychological issues
Schwarz et al. 2012 [53]	Home care	Cardiologist, palliative physician, Family doctor, nurse, social worker	*Experience*: pain and anxiety improved, less use of opioids; increased clarity about treatments; improved management	Psychological issues Relationship with professionals or services;
Harding et al. 2008 [61]	Home care	Cardiologist, palliative physician, nurse	Experience: confusion of HF and symptoms; no discussion about future; anxiety, anger and confusion experienced by patients and perceived by FCGs; not easy to talk with doctors (too busy) Expectations: need to know more about future; easy language that can be understood; family conferences with staff, provide a support group or phone line	Relationship with professionals or services; Worries and plans for the future; Relationship with patients
Ng et al. 2017 [73]	Home care	Cardiologist, palliative physician, nurse	*Experience*: focus on physical and emotional health, social life, financial status, and the relationship between caregiver and patient. Significant decrease of burden in control group	Psychological issues; Relationship with patients; Financial aspects
Im et al. [70]	Home care	Cardiologist, palliative physician, nurse, social workers	Experience: good awareness of illness management, lack of awareness about future, no talking about death, not engaged in end of life discussion	Relationship with professionals or services; Worries and plans for the future; Impact of patients’ condition on FCGs; Relationship with patients
Ross et al. 2015 [63]	Home care	Cardiologist, palliative physician, nurse, volunteers, chaplain	Experience: struggle for incongruence of view about medications and general issues; feeling isolated; importance of support from religious leaders Expectations: creating care co-ordinator, voluntary organizations, support groups, home visiting services. Link with chaplaincy team in hospital	Psychological issues
Hupcey et al. 2011 [50]	Home care	Nurse	Experience: financial matters like lost work/absence from work, cost of travel for visits, medication expenses; psychosocial issues like family conflict increase, role change; physical issues like unaddressed medical needs, stress, health problem for FCGs Expectations: easily understandable information regarding treatment options, advanced directives, future planning	Psychological issues; Financial aspects
Brannstrom et al. 2007 [72]	Home care	Nurse, physioterapist	Experience: Feeling secure through getting help and support from the team members, tailored-care at home to manage everyday life; feeling isolated at home, physically burdened, burdened by responsibility, constantly worried; anxiety and uncertainty Expectations: being consoled for loved one’s suffering, relief in meeting someone (team) to share and ease their burden	Psychological issues;
Neuwirth et al. 2012 [49]	Home care	Nurse, social worker, pharmacist	Experience: helpful communication between patients, caregiver and healthcare providers; video effective in reducing re-hospitalization	Reletionship with professionals or services
Boyd et al. 2004 [60]	Home care	Palliative physician, Family doctor	Experience: comorbidities as a huge problem, frustration conveyed to carers; low mood and anxiety; carer left alone and not recognized as a key figure; passive role in decision-making; difficulty in decision due to uncertainty of prognosis and difficulty in discussing it Expectations: home visiting, better coordination in hospital; extending the role of PC services	Psychological issues; Impact of patients’ condition on FCGs
Dionne-Odom et al. 2014 [55]	Home care	Palliative physician, nurse	Experience: FCGs burned out; intervention helped but many already know how to manage things; helpful to have a nurse coach; Expectations: intervention would be better earlier	Psychological issues; Impact of patients’ condition on FCGs Relationship with services/profess
Mcmillan et al. 2007 [56]	Hospice	Cardiologist, palliative physician, nurse, social worker	Experience: little social support; some carers expressed depressive symptoms Expectations: more focus on depressive symptoms	Psychological issues; Impact of patients’ condition on FCGs
Buck et al. 2013 [48]	Hospice	Palliative physician, nurse	Experience: informative tools are perceived as useful to understand things for those with few weeks of caregiving, useless for long stays; useful for diagnosis; hospice (with nurses) as a solution to solve everyday problems and to focus on spiritual issues Expectations: identify specific groups of FCGs (newer, hospice FCGs); offering the intervention earlier; discussing symptom management in a multimorbid setting	Relationship with professionals or services Role of pc
Metzger et al. 2013 [52]	Hospital	Cardiologist, palliative physician	Experience: unprepared for PC consult, no previous understanding of PC; suspicion, caution; PC was welcomed for those who know; general positive effect of PC team; role of support for PC emotional state and for managing care, meetings, obstacles, providing information; Conflation of PC and hospice as a barrier to PC	Relationship with professionals or services; Role of PC
Browne et al. 2014 [62]	Hospital	Cardiologist, palliative physician, Family Doctor, nurse, other specialists, pharmacist	Experience: poor understanding of treatments; PC and hospice only for few patients; services not coordinated, lack of communication between different professional figures Expectations: more communication, coordination between all people involved	Relationship with professionals or services
Metzger et al. 2013 [54]	Hospital	Cardiologist,palliative physician	Experience: no awareness of PC; no aggressive measures for symptoms; hospice seen as death imminent; resistant to PC for those who didn't know; for other, PC is welcomed Expectations: true understanding of PC and hospice	Role of PC
Alonso et al. 2017 [45]	Hospital and hospice	Cardiologist, palliative physician	Experience: not all perceive disease severity; significant relationship between perceiving illness severity and PC service utilization Expectations: more understanding of disease severity	Impact of patients’ condition on FCGs
Small et al. 2009 [66]	Hospital, home care, nursing home	Cardiologist, nurse	Experience: lack of communication with professionals; no discussion on the place of death and how to die; difficulty to discuss; importance of faith and religion. Perception of death in home, sudden or in nursing home as a "good death" generally seen peaceful; complaints on hospital care, too many unnecessary interventions, unsympathetic staff; wish for "making comfortable" approach for patients; lack of bereavement support	Psychological issues; Relationship with professionals or services
Chester et al. 2021 [67]	Hospital, Hospice, Home care	Cardiologist, nurse	Experience: awareness of diagnosis, variable understanding of disease severity; feer associated with palliative care; no understanding of transfer to hospice; positive experience of shared decision-making process between patient, carer and healthcare professionals; pivotal role of advanced nurse practitioner—perceived as a broker in a complex health and social care system	Relationship with professionals or services; Worries and plans for the future; Role of PC
Alvariza et al. 2017 [71]	LTC facilities	Cardiologist, nurse	Experience: happiness, trust in healthcare professional care; did not need support for themselves, feelings of relief from sharing responsibility with providers; feeling of isolation, the main focus was on patients; lack of communication with providers	Psychological issues; Impact of patients’ condition on FCGs Relationship with professionals or services
Kaasalainen et al. 2013 [68]	LTC facilities	Cardiologist, nurse, social worker, pharmacist	Experience: difficulties in understanding HF related problems and those related to other diseases; unpredictable and sudden HF exacerbations; lack of communication between patients, CG and providers. Nurses were the key figures to coordinate with; social workers seen as helpful (sometimes they lack relevant knowledge) Expectations: need more info about HF and strategies of care; need for individualized care; need for family support; need to have specialist consultation outside LTC	Relationship with professionals or services; Worries and plans for the future; Impact of patients’ condition on FCGs

Hospital was the place of care in three studies [52, 54, 62], where teams included palliative specialists and cardiologists, or a larger multiprofessional team [62]. Two studies had hospice as the place of care [48.56], and in Mcmillan et al. [56] palliative professionals and nurses worked together with cardiologists and social workers. Two studies from Sweden and Canada [66, 68] had long term care (LTC) facilities as the setting of care and palliative specialists were not involved in the care team. Finally, when mixed setting of care (hospital, home care and nursing home; hospital and hospice) [45, 66, 67] were present, patients were supported by heterogeneous professional teams. A few studies reported whether patients experienced care transitions (for example, patients moved from the inpatient PC unit and inpatient hospice) [54], or there was a reference to how professionals approached the transition to PC [59].

### FCG experiences

We grouped the experience of FCGs of advanced HF patients in by seven topics.

- *1) Psychological issues* (48.4%). FCGs reported: negative feelings [57, 63, 66, 72, 74], such as emotional strain, psychosocial problems, depression, anxiety, isolation, struggling; relief and happiness from sharing responsibility with providers and relieving their mental struggles, pain and anxiety [53, 57, 71].
- *2) Relationship with professionals or services* (51,6%). FCGs experienced lack of relationship and communication with physician lack of services in the community, difficulties contacting the hospital, and unsympathetic staff [62].
- *3) Worries and plans for the future* (22,6%). They dealt with: unpreparedness and fear for the future [44, 70]; unclear understanding of the disease or treatment [45, 59, 61, 67, 68, 74], as well as symptoms; unwillingness to talk about death [59, 70]; frustration and sadness for their exclusion from the patients’ plans about their death.
- *4) Impact of patient condition on FCGs (38,7%).* They mainly reported a negative impact on daily life [46, 57], and the consequent impossibility of going out as usual, stress and challenges in everyday life, isolation at home, curtailed daily activities, and changes in dyad roles.
- *5) Relationship with patients (19,4%):* FCGs saw patients negatively approaching end-of-life issues [74], such as struggling with symptoms and being unwilling to talk about death. One study only reported common feelings and views of patients and FCGs on managing the disease [51].
- *6) Financial aspects (6,5%):* FCGs reported financial problems due to missing work or costs for travel and medications [50].
- *7) Role of PC (16,1%):* in some cases, it is common among FCGs to lack awareness or understanding of PC services [52, 54] and, in turns, experiencing resistance to access to PC for families that did not know about PC [54]; sometimes, the presence of PC specialists improved the care management.

### FCG Expectations

Many studies highlighted FCGs’ expectations in terms of the following:

- *1) Worries and plans for the future* (22.6%): FCGs needed more information about future prognosis and the disease [45, 46, 50, 58, 61] and, specifically, having access to easily understandable information regarding treatment options and future planning [58].
- *2) Relationship with professionals or services* (12.9%): they expected improvements in communication with professionals [50, 62] to ease their burden better and foster better coordination between professionals joining the multidisciplinary teams.
- *3) Psychological issues* (19.4%): due to their need to receive support in groups [61, 63], by accessing family conferences or phone lines to improve patient care management or with more attention on depressive symptoms.
- *4) Role of PC* (9.7%): FCGs asked for prompt initiation of PC interventions or, sometimes, they needed a better understanding of PC [54].
- *5) Impact of patient conditions on FCG* (3.2%) daily life: FCGs need more support in managing the burden of their family member’s disease on their daily life [68]; the impact of patient conditions moves FCGs to sacrifice their life for them, reducing time the FCG can spend on their activities while generating needs for spiritual support.

### Experiences and expectations according to the setting of care and provider profile

**Fig. 2:**
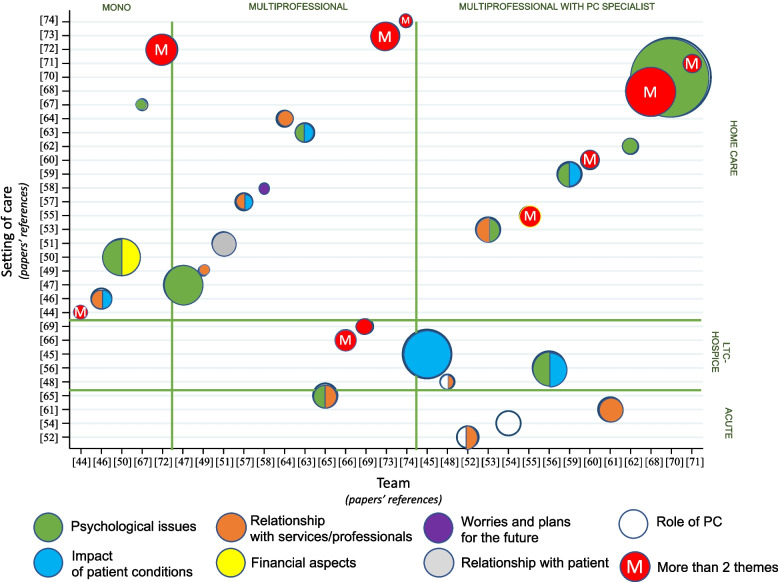
Map of FCG experience topics by care setting and care team composition. *Note*: (**a**) the size of the circles refers to the number of FCGs enrolled in each study; (**b**) bicolour circles refer to two topics, red circles refer to multiple topics, (**c**) axis numbers refer to the reference number of the reviewed papers

Table 4 highlights FCG experiences and needs disaggregated by the description of the place of care and the health/social professionals involved in delivering care. Figure 2 maps the occurrence of the seven topics across studies that we grouped on the y-axis by setting of care (acute, LTC-hospice, homecare) and on the x- axis by compositions of care team(s) (mono-professional, multiprofessional and multiprofessional with PC specialist). Each circle represents a study, its color refers to a topic (as detailed in the figure legend), and the size of the circle indicates the number of FCGs involved in the study.

When we investigated any differences in FCG experiences due to the combination of setting of care and care provider (Fig. 2), we observed that these differences may have been related to the professional team responsible for the patient care or to the setting of care. In LTC-hospices and homecare, mainly when the support of a PC specialist was absent, FCGs reported “worries and plans for future” [44, 59, 67, 68, 74]. A difficult “relationship with patients” were reported when the setting of care was the home, regardless of the team composition [51, 57, 61, 70, 73, 74]; in a few cases, a palliative physician was dedicated to FCG relationship management [56, 61, 62]. Comments on “Relationship with professionals” were, in proportion, more frequent in acute and longterm care; “psychological issues” and “impact of patient conditions” on their lives, were more recurrent in multi-professional teams, also when PC specialist were involved [45, 53, 55, 56, 60, 63, 69, 70, 73].

FCGs of patients assisted by professionals who worked as “brokers” across services reported positive opinions about the management of the end-of-life period; specifically they obtained information about disease and treatments and experienced prompt management of their negative feelings [67].

The management of “worries and plans for the future” was experienced independent of the team composition [44, 58, 59, 61, 67, 68, 70, 74]; the need for more communication (“relationship with professionals”) was a transversal topic. Even when patients received care from a multiprofessional team with a palliative physician, FCGs asked for an earlier PC intervention [55].

## Discussion

A recent review [52] made a comprehensive assessment of the state of the art in family caregiving of HF patients but did not focus on who is in charge of patient care and where care is provided. To the best of our knowledge, this is the first literature review on the experience of FCGs of end-stage HF patients by investigating whether and how their experiences vary across the setting of care and according to the professional profile of the care team. It is important to understand how patients’ and families’ outcomes and satisfaction with end-of-life care may be affected by structural and professional factors of service delivery to support the redesign of care pathways in the future [75].

The papers we analysed were mainly from the USA, UK and Sweden and reported psychosocial issues and relationships with patients and professionals as fundamental topics for the FCGs of HF patients at the end-of-life. These findings are consistent with data from other reviews [43]. The lack of a “relationship with professionals” turns out to be a key factor in understanding why FCGs experienced uncertainty and unawareness of the prognosis and future. Imes and colleagues [46] observed that the lack of information from healthcare providers increased frustration in FCGs. Generally, the topic of unmet needs about the relationship with healthcare professionals was addressed in several studies, as reported in a recent review [76]. We also observed positive experiences from FCGs dealing with the “management of negative feelings”.

Depending on the setting of care and professional profile, we observed some differences in FCG experiences and expectations. Feelings such as “worries and plans for the future” were mainly reported when HF patients were assisted in LTC-hospice and home care settings and when they did not receive care from palliative specialists. Additionally, there were perceptions of a negative impact on daily “relationships with patients” when patients were treated at home. Depression, emotional strain, isolation, and anxiety (“psychological issues”) were recurrent among FCGs when the care environment coincided with the home and when FCGs could not benefit from the physical and emotional home rest when moving from healthcare facilities to the home. These issues were not particularly present when professionals, perceived as brokers, were responsible for patient care [67]. Conversely, studies reported positive experiences among FCGs when the settings of care were LTC facilities, hospices, or hospitals, as well as when the home care landscape was combined with a “complete” multidisciplinary team composed of, for example, family doctors, cardiologists, palliative physicians and nurses. The latter findings were also previously observed by Fendler and colleagues [77].

Based on the above evidence, it is possible to hypothesize that there is room for improvement in the management of end-of-life care with regard to FCGs’ feelings and perceptions. This could be addressed by the adoption of a care model that clearly and adequately promotes PC in a timely fashion, involves a multidisciplinary team who drive patients and FCGs through the pathway and the healthcare systems ‘ services, and identifies the most adequate setting of care for patient and FCG needs. The benefits provided by multidisciplinary teams, also confirmed for end-stage HF patients, are generally known [77]. Specifically, the involvement of professionals such as palliative doctors and social workers can have a positive impact on FCGs’ lives and experiences of care. Palliative doctors can support FCGs in facing negative feelings and perceptions and mediating daily relationships between patients and FCGs [77], and social workers can play a positive role in communication and coordination processes [78]. Accompanying FCGs in this critical journey and making FCGs confident in facing most of the daily socioeconomic and health matters can lead FCGs to feel “taken in charge”, with the positive effect of helping them to better manage their anxieties and, in turn, to reduce inappropriate accesses to acute care services.

*Implication for clinical practice:* This review provides significant insights about the role of PC. PC has a pivotal impact on positive and negative FCG experiences: there were FCGs who believed in the benefits of PC treatments, but sometimes complained of a lack of prompt provision; most frequently FCGs were under-informed of PC and their unawareness on the existence and benefits of PC could cause them to ask for other and more inappropriate access to healthcare services.

The evidence from our literature review moves towards clear recommendations for offering patients and FCGs the support of a complete multidisciplinary team, that adopts processes and roles that are well defined and clearly communicated with patients and FCGs. It is ideal for cardiologists and palliative care providers to work together to achieve common goals for the wellbeing of patients and their relatives.

*Policy recommendations* – There are still few policies on the transition from interventional to PC for chronic patients and insufficiently widespread national programs to support FCGs care for HF patients at the end of life [79]. When available, national guidelines are not completely applied at the operational level, causing poor coordination between acute and palliative units [80] and producing a negative effect on both patients and FCGs. Nevertheless, the needs of FCGs are key and fundamental issues and taking care of them can lead to reducing the burden of disease for patients and their relatives, to improve experience of care and quality of life and to provide appropriate and efficient responses by healthcare systems. Hence, findings of our review may inform policymakers and healthcare managers to modify the end of life services they provide. Finally, the improvement of FCG experiences can help to increase patient access to PC.

However, nowadays the provision of PC by PC specialists is not possible in many countries, also due to local barriers. In those cases, policies and regulations must additionally propose context-based and personalized solutions to satisfy the needs of patients and FCGs based on continuous listening processes that involve patients, FCGs, healthcare professionals and services managers.

### Limitations

Our review has several limitations. First, the research was limited to papers indexed in PubMed, Scopus and Web of Science; thus, there may be articles that suited the research flowchart that were missed. Furthermore, research criteria included articles from 2000 to 2021, which was a very large timeframe in which many things changed in HF and PC in terms of medical therapies, cultural beliefs and policies, particularly the impact of the COVID-19 pandemic. Additionally, the knowledge cannot be easily generalized because the studies were qualitative in nature, because their results have been extrapolated from local settings that can differ greatly and because there were too few studies for each context to fully represent the country where the study was conducted. In addition, the majority of papers came from the USA, UK and Sweden, countries in which PC in HF is widespread and where many resources are available for its development.

## Conclusions

FCGs have a crucial role in HF and end of life care management. It has been estimated that the burden of informal caregiving for patients with cardiovascular diseases will rise in the next 20 years [81]; consequently, health systems must pay attention to FCG needs. This review shows that depending on the setting of care and professionals involved, the existing services fulfil FCG needs and expectations in different ways; in addition, it confirms that national health systems adopt heterogeneous models of care across the world. The results show that there are no relevant differences in terms of FCG experience across the settings of care. The main exception refers to the home setting, where there is a larger experience of psychological issues than in hospital, hospice and LTC. Instead, FCGs’ feelings and perceptions of the burden of disease on their lives seem to vary depending on the presence or the lack of specific professionals within the team of care, such as palliative doctors and social workers.

## Supplementary Information

**Additional file 1:Supplemental 1.** Algorithm of search

## Data Availability

All articles analyzed in this literature review are listed in the Reference section, and can be accessed coherently with the access policy of the publisher.

## Abbreviations

FCGs: Family caregivers
HF: Heart failure
PC: Palliative care
