# Supplementary material for: Differences in family caregiver experiences and expectations of end-of-life heart failure care across providers and settings: a systematic literature review
Source: BMC Health Serv Res. 2023 May 3;23:429. doi: 10.1186/s12913-023-09241-w (PMC10155156; doi:10.1186/s12913-023-09241-w)
Supplement: Supplementary file 1 — Additional file 1:Supplemental 1. Algorithm of search [file 12913_2023_9241_MOESM1_ESM.docx]

Supplemental 1. Algorithm of search

| Electronic Database | Algorithm |
| --- | --- |
| Pubmed | (((((("heart failure"[Title/Abstract] OR "cardiac failure"[Title/Abstract] OR "congestive heart failure"[Title/Abstract] OR "advanced heart failure"[Title/Abstract])) AND ("terminal care"[Title/Abstract] OR "end of life"[Title/Abstract] OR "palliative care"[Title/Abstract])) AND (caregiver*[Title/Abstract] OR carer*[Title/Abstract] OR caregiving*[Title/Abstract] OR famil*[Title/Abstract] OR relatives[Title/Abstract]))) NOT review[Publication Type]) |
| Scopus | TITLE-ABS-KEY ( "Heart failure" OR "cardiac failure" OR "advanced heart failure" ) AND TITLE-ABS-KEY ( "end of life" OR "palliative care" ) AND TITLE-ABS-KEY ( caregiver OR caregivers OR carer OR carers OR caregiving OR families OR family OR relatives) AND NOT DOCTYPE ( re ) |
| Web of Science | TOPIC ("Heart failure" or "cardiac failure" or “congestive heart failure” or "advanced heart failure") AND TOPIC: ("palliative care" or "end of life" or “terminal care”) AND TOPIC: (carer* or caregiver* or cargiving or famil* or relavities) NOT DOCUMENT TYPES: (Review) |
